# *Salmonella typhimurium*-induced IL-1 release from primary human monocytes requires NLRP3 and can occur in the absence of pyroptosis

**DOI:** 10.1038/s41598-017-07081-3

**Published:** 2017-07-31

**Authors:** Catherine E. Diamond, Keith Weng Kit Leong, Maurizio Vacca, Jack Rivers-Auty, David Brough, Alessandra Mortellaro

**Affiliations:** 1 Singapore Immunology Network (SIgN), Agency for Science, Technology and Research (A*STAR), Singapore, 138648 Singapore; 2 Division of Neuroscience and Experimental Psychology, Faculty of Biology, Medicine and Health, Manchester Academic Health Science Centre, University of Manchester, Manchester, M13 9PT UK

## Abstract

Large molecular complexes known as inflammasomes regulate the release of IL-1β from immune cells in response to infection and injury. *Salmonella typhimurium* infection is reported to activate NLRP3 and NLRC4 inflammasomes which are subsequently involved in pyroptosis of the cell and pathogen clearance. However, the response to *S. typhimurium* in primary human monocytes has not been studied in detail. The aim of this study was to investigate the effect of *S. typhimurium* on inflammasomes in primary human monocytes. Much of the previous research in the field has been conducted in murine models and human THP-1 cells, which may not reflect the responses of primary human monocytes. Here, we report that inhibiting NLRP3 with the selective inhibitor MCC950, blocked release of IL-1β and the related cytokine IL-1α from primary human monocytes in response to *S. typhimurium*. Additionally, under these conditions *S. typhimurium*-induced IL-1 release occurred independently of pyroptosis. We propose that IL-1β release without pyroptosis may occur in early-recruited monocytes to regulate a maximal innate immune response to *Salmonella* infection, allowing a sustained inflammatory signal. This insight into the mechanisms involved in IL-1 release from primary human monocytes highlights major differences between immune cell types, and the defences they employ during bacterial infection.

## Introduction

*Salmonella enterica* serovar Typhimurium (*S. typhimurium*) is a food and water-borne, Gram-negative bacteria that causes millions of cases of gastroenteritis, fever and septicaemia a year, particularly in infants and young people. However, infection can often be avoided by an understanding of food hygiene and sanitation. With the increasing prevalence of antibiotic resistance, fatalities from *S. typhimurium* infection are becoming more common^1, 2^. Therefore, it is critical to understand the immunological mechanisms conferring protection against *S. typhimurium* infection.

Primary human monocytes are crucial for the innate immune response to *S. typhimurium* infection. During salmonellosis, the speed of recruitment of blood monocytes to peripheral tissues is greatly increased, aiding the clearance of bacteria^3^. This early response to infection is vital as tissue resident cells cannot clear the infection without recruitment of neutrophils and monocytes which can differentiate into macrophages and dendritic cells within tissues^4^. During the acute stages of microbial infection monocytes release cytokines. Cytokines regulate the clearance of infection from the host by co-ordinating the recruitment of cells, and the distribution and response of different cell types. Two important cytokines are members of the interleukin-1 (IL-1 family) IL-1α and IL-1β^5, 6^. IL-1α and IL-1β are produced as precursors (pro-forms) that require cleavage to mature forms before being released from the cell to activate the type I IL-1 receptor (IL-1RI). Our current understanding suggests that IL-1α and IL-1β utilise different mechanisms to regulate their post-translational maturation and secretion^7^. The transcription of pro-IL-1α and pro-IL-1β is upregulated when a pathogen associated molecular pattern (PAMP), such as lipopolysaccharide (LPS), is sensed by a pattern recognition receptor (PRR), such as Toll-like receptor (TLR) 4. This initial induction of IL-1 gene expression is referred to as a priming step. In order for IL-1β to be secreted, the protease caspase-1 also needs to be activated before it can cleave pro-IL-1β^8, 9^. This process requires the formation of large multi-molecular protein complexes known as inflammasomes. These are assembled when an additional signal, often a damage-associated molecular pattern (DAMP), or another PAMP is detected. Inflammasomes contain cytosolic PRRs including the members of the nucleotide-binding oligomerization domain (NOD)-like receptors-like receptor (NLR) family^10^. NLRP3 (NACHT, LRR and PYD domains containing protein 3) is the most extensively studied NLR and forms an inflammasome by associating with the adaptor protein ASC (apoptosis-associated speck-like protein containing a CARD). This in turn recruits and then induces the auto-activation of caspase-1, leading to the cleavage and secretion of IL-1β^11^. In contrast to IL-1β, pro-IL-1α can bind to IL-1RI without cleavage. However, after processing by proteases into its mature form, IL-1α is more active at IL-1RI^12, 13^. Primary human monocytes have been shown to have the ability to circumvent this two-signal inflammasome dependent pathway for release of IL-1β, required by other immune cells such as monocyte-derived macrophages, and can employ a one-step pathway to secrete mature IL-1β^14^.

*S. typhimurium* can introduce LPS into the intracellular environment as it contains type III secretion system apparatus. Once within the cytosol, LPS is detected by caspase-11 in mice, or in human cells by caspase-4 and caspase-5, which then induce the non-canonical NLRP3 inflammasome pathway^15^. Translocation of *S. typhimurium* into the cytosol can also activate the NLR containing card domain 4 (NLRC4) inflammasome, through co-oligomerisation with NAIPs (the NLR family of apoptosis inhibitory proteins)^16^. NAIPs are cytosolic detectors of the structural components of type III secretion system of Gram-negative bacteria^17^. Unlike NLRP3, NLRC4 contains a CARD domain that can directly interact with pro-caspase-1, without the necessity for an ASC adaptor, however the cleavage of IL-1β still requires ASC^18^. Recent studies have shown that both NLRP3 and NLRC4 play a role in inflammasome activation during the clearance of the pathogenic Gram-negative bacterium *S. typhimurium* ^16, 18^.

*S. typhimurium*-induced activation of caspase-1 causes an inflammatory form of programmed cell death known as pyroptosis in macrophages^19, 20^. It is unknown if monocytes undergo pyroptosis in response to *Salmonella* infection. Much of the previous research into the inflammasome activation pathway in response to *S. typhimurium* has been conducted in murine models or in the human monocytic leukemic THP-1 cell line. However, we and others previously reported important differences in the mechanisms of NLRP3 inflammasome activation between human monocytes and cell lines^14, 21^, highlighting specific responses of different immune cell types.

The aim of this study was to investigate the extent to which *S. typhimurium* induced IL-1 production is dependent on NLRP3 in primary human monocytes, and the association between cytokine production and pyroptosis in response to *S. typhimurium*. In response to *S. typhimurium* infection, the release of IL-1 was completely blocked by the specific NLRP3 inhibitor MCC950^22^. Further, we show that under the conditions reported here the release of IL-1β and IL-1α by human blood monocytes was independent of pyroptosis. These data suggest that in primary human monocytes following *S. typhimurium* infection IL-1β release requires NLRP3 and can occur independently of pyroptosis.

## Results

### MCC950 inhibits IL-1β and IL-1α release from primary human monocytes

**Figure 1:**
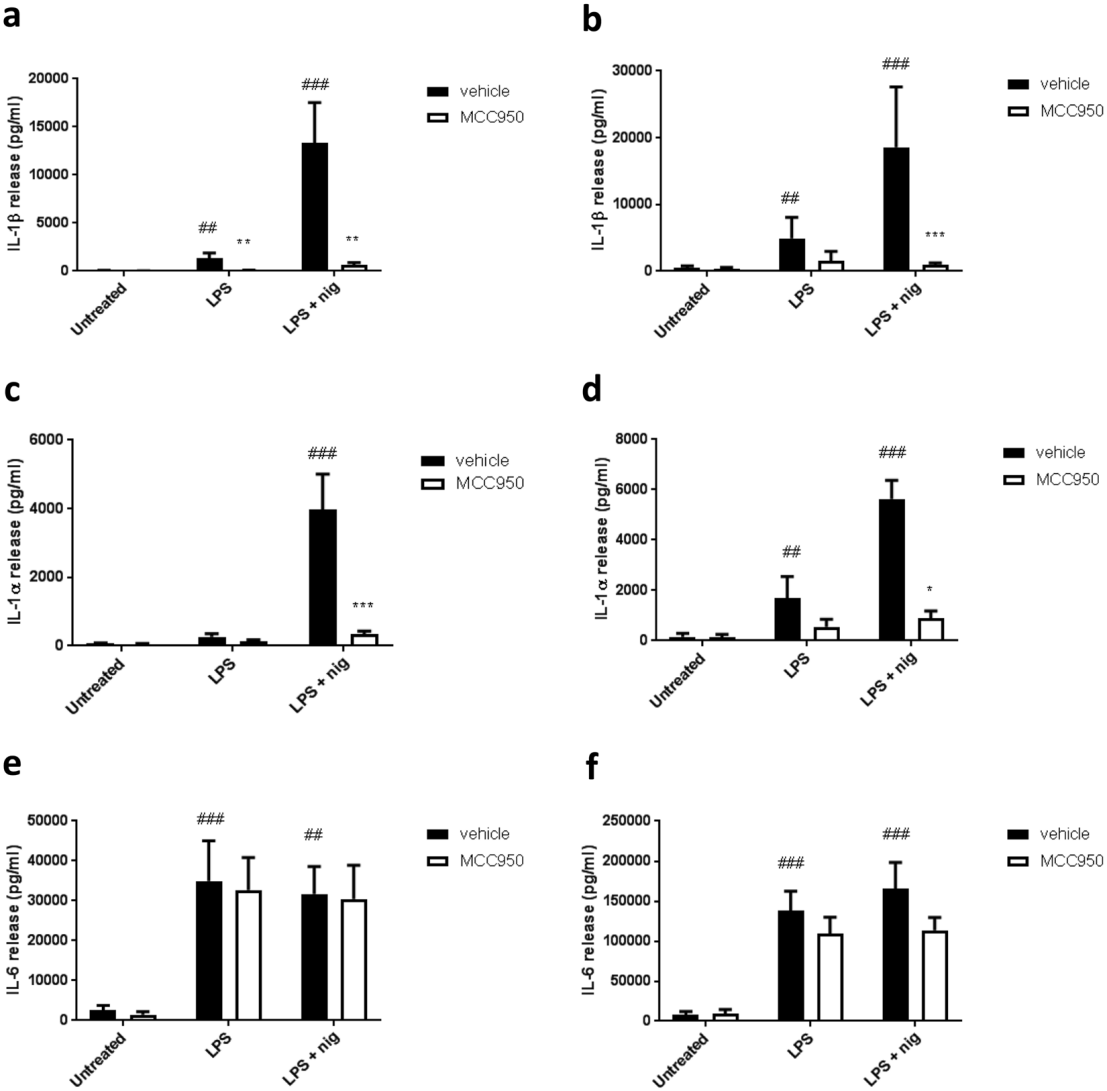
NLRP3 is required for the release of IL-1β and IL-1α from primary human monocytes. Primary human monocytes were either pre-treated with MCC950 (10 μM) or not, and then stimulated with LPS (1 μg/ml) for 4 h. Nigericin (10 μM) or vehicle was added for 1 h after LPS incubation. Response was measured by IL-1β (**a**,**b**), IL-1α (**c**,**d**), and IL-6 (**e**,**f**) release after 5 h incubation (**a**,**c**,**e**) and overnight incubation (**b**,**d**,**f**), as assessed by ELISA. Data are presented as mean cytokine release ± s.e.m. Graphs (**a**,**c**,**e**) are representative of 4 independent donors and graphs (**b**,**d**,**f**) representative of 5 independent donors, each performed in triplicate wells. *P < 0.05, **P < 0.01, ***P < 0.001 significant effect of MCC950 compared to vehicle group with matching stimuli. ^#^P < 0.05, ^##^P < 0.01, ^###^P < 0.001 significant induction of cytokine release by stimuli compared to untreated control. Significance measured with a with-in subjects design two-way ANOVA followed by Holm-Šidák corrected post-hoc analyses.

The NLRP3 inhibitor MCC950 inhibits IL-1β release^22–24^, and so MCC950 was used to investigate whether NLRP3 was important for IL-1 release from primary human monocytes. Isolated primary human monocytes received either vehicle (DMSO), or MCC950 (10 μM), for 30 min prior to priming with LPS for 4 h or overnight. The cells were then stimulated with the K^+^ ionophore and NLRP3 inflammasome activating stimulus nigericin for 1 h. MCC950 treatment significantly inhibited IL-1β release in LPS primed cells in response to nigericin at both the 5 h and overnight time points (Fig. 1a,b). Primary human monocytes also release IL-1β in response to LPS alone and this was also significantly inhibited by MCC950 (Fig. 1a). MCC950 also inhibited nigericin-induced IL-1α release from LPS-primed cells (Fig. 1c,d). As with IL-1β, there was some IL-1α release to LPS alone and MCC950 significantly inhibited this (Fig. 1c,d). LPS-induced release of another pro-inflammatory, but NLRP3 inflammasome independent cytokine, IL-6 was unaffected by MCC950, demonstrating that MCC950 specifically inhibited the NLRP3 inflammasome.

### Intracellular LPS causes NLRP3-dependent IL-1β release

**Figure 2:**
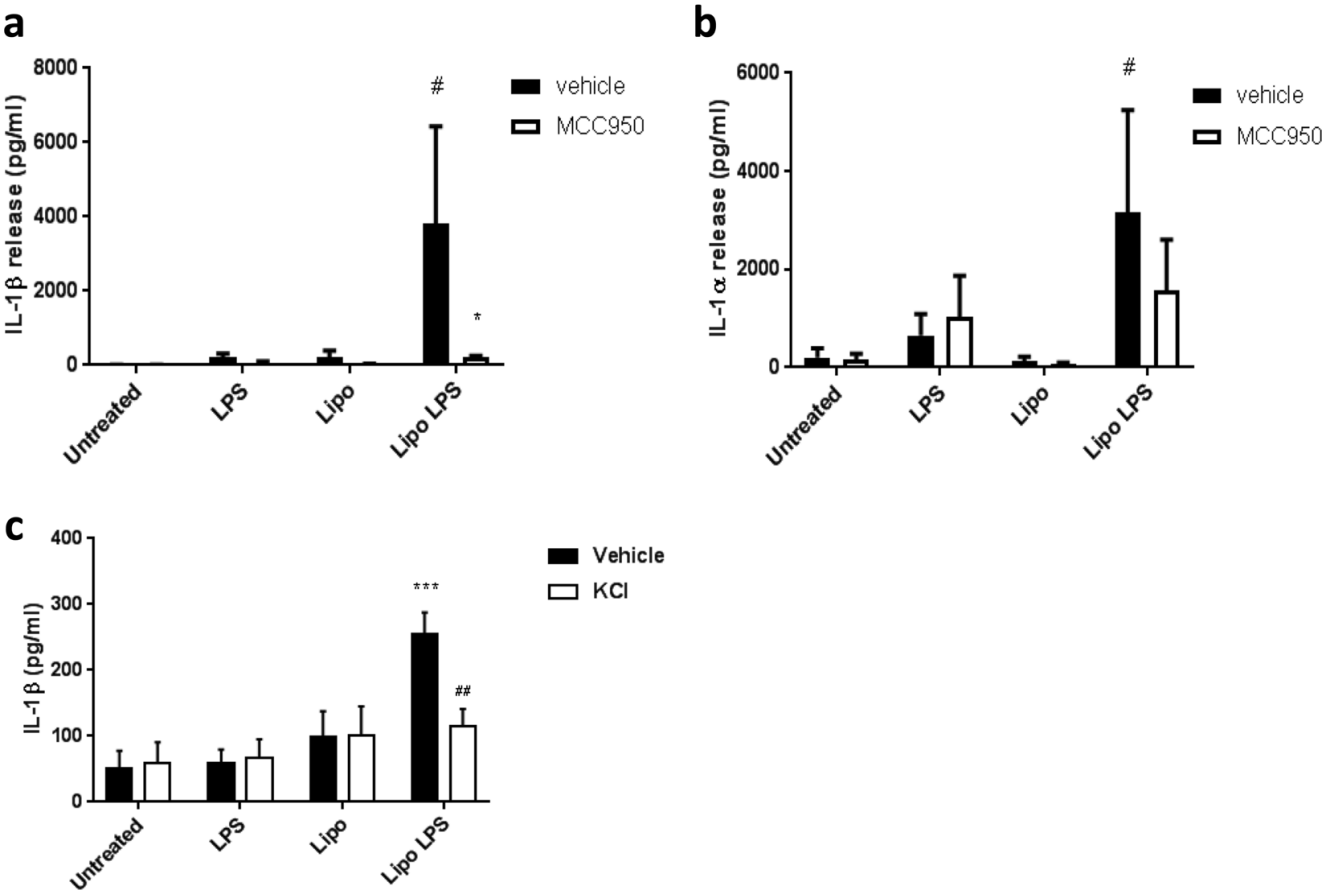
MCC950 causes a decrease in IL-1β, but not IL-1α, release following the activation of the non-canonical inflammasome pathway. Primary human monocytes were either pre-treated with or without MCC950 (10 μM) (**a**,**b**), or in the presence or absence of extracellular KCl (20 mM) (**c**). LPS was then transfected into the monocytes using Lipofectamine 2000 (Lipo LPS), or added extracellularly without Lipofectamine (LPS). Lipofectamine without LPS was used as a control (Lipo). Response was measured by IL-1β (**a**,**c**) and IL-1α (**b**) release into cell supernatant by ELISA. Data are presented as mean cytokine release ± s.e.m. Graphs are representative of three independent donors, each performed in triplicate wells. **P < 0.0101, ***P < 0.001 significant effect of MCC950 compared to vehicle group with matching stimuli. ^#^P < 0.05, ^##^P < 0.01 significant induction of cytokine release by stimuli compared to untreated control. Significance measured with a with-in subjects design two-way ANOVA followed by Holm-Šidák corrected post-hoc analyses.

Cytosolic LPS activates the non-canonical NLRP3 inflammasome pathway^24, 25^. To determine if both IL-1β and IL-1α release were dependent on NLRP3 in response to activation of the non-canonical pathway, human primary monocytes were treated with or without MCC950 (10 µM, 30 min) as described above, then transfected with LPS using Lipofectamine, an established model for activating the non-canonical inflammasome^24^. Transfected LPS (Lipo LPS) induced IL-1β secretion from human monocytes, which was almost completely abolished by MCC950 (Fig. 2a). Conversely, IL-1α release was not significantly reduced by MCC950 (Fig. 2b). These data suggest that IL-1β secretion following activation of the non-canonical pathway is NLRP3 dependent, while IL-1α release is not. Recent research suggested that the non-canonical NLRP3 activation pathway required an efflux of K^+^^26^, and in our experiments NLRP3-dependent IL-1β release in response to LPS transfection was inhibited by raised extracellular K^+^ (Fig. 2c).

### Release of IL-1β and IL-1α is dependent on NLRP3 in response to acute *S. typhimurium* infection in primary human monocytes

**Figure 3:**
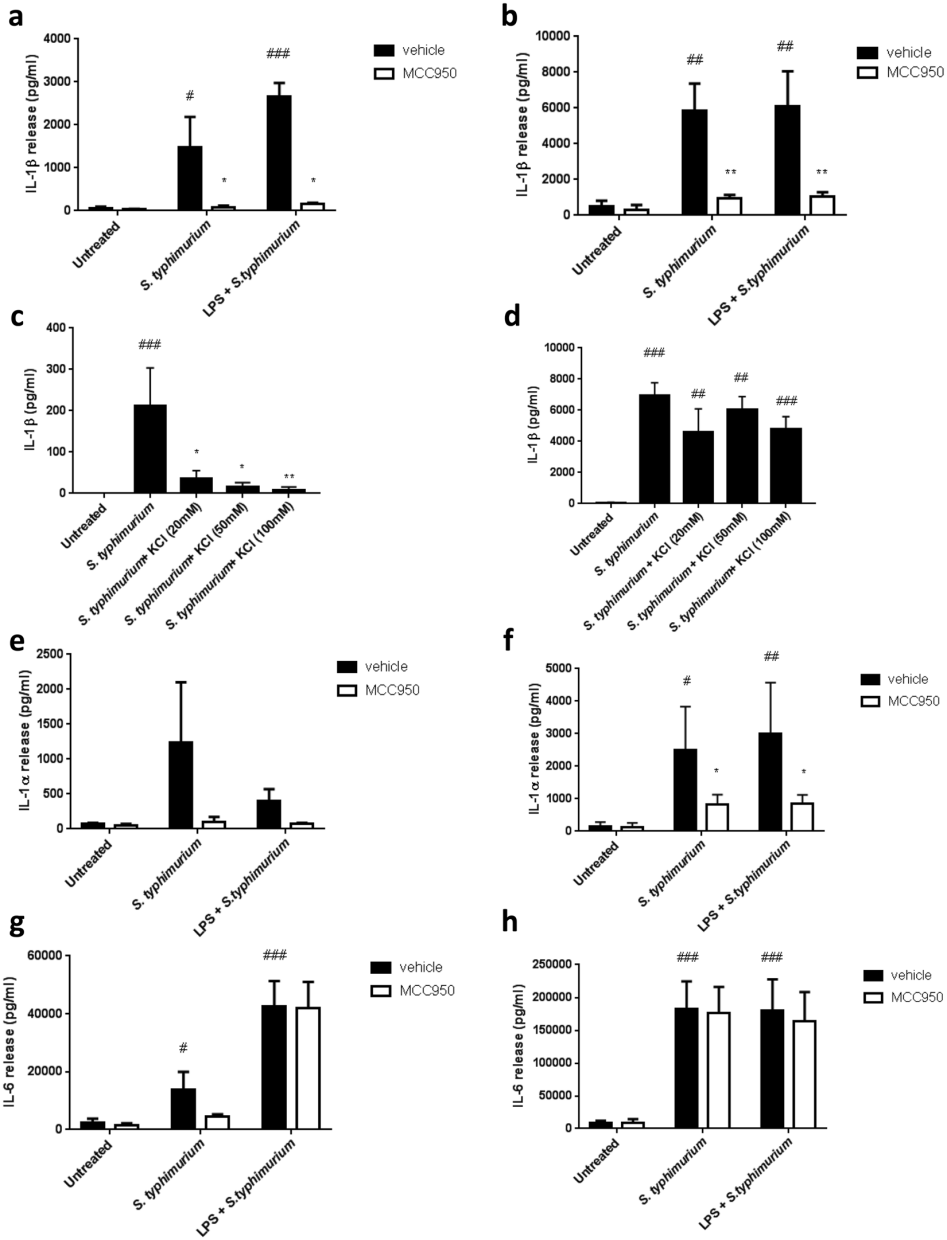
Efficient release of IL-1β and IL-1α is dependent on NLRP3 in response to acute *S. typhimurium* infection, and LPS priming is not required for *S. typhimurium* induced IL-1β release. Primary human monocytes were either pre-treated with or without MCC950 (10 μM), and then incubated for 3 h with or without LPS (200 ng/ml). *S. typhimurium* (MOI 10) was added 1 h before addition of gentamycin and further incubation of either 1 h (**a**,**c**,**e**,**g**) or overnight (**b**,**d**,**f**,**h**) in the presence and absence of extracellular KCl (20, 50, 100 mM) (**c**,**d**). Response was measured by IL-1β (**a**–**d**), IL-1α (**e**,**f**), and IL-6 (**g**,**h**) release into cell supernatant by ELISA. Data are presented as mean cytokine release ± s.e.m. Graphs (**a,c,d,e,g**) representative of four independent donors and graphs (**b**,**f**,**h**) representative of 5 independent donors, each performed in triplicate wells. *P < 0.05, **P < 0.01 significant effect of MCC950 or KCl treatment compared to vehicle group with matching stimuli. ^#^P < 0.05, ^##^P < 0.01, ^###^P < 0.001 significant induction of cytokine release by stimuli compared to untreated control. Significance measured with a with-in subjects design two-way ANOVA followed by Holm-Šidák corrected post-hoc analyses.

It is currently unclear if NLRP3 is required for IL-1 release from primary human monocytes in response to *S. typhimurium*. Recent evidence has suggested that the NLRP3 has a limited role in the clearance of *S. typhimurium in vivo* in mice^27^, and inhibiting NLRP3 did not affect IL-1β release *in vitro* in bone marrow-derived macrophages (BMDMs) after *Salmonella* infection^22^. It was also suggested that both NLRP3 and NLRC4 contribute towards the innate immune response to *S. typhimurium*, as NLRP3 is recruited by NLRC4 in order to amplify the caspase-1 response^18, 28^. MCC950 was used to test whether IL-1 release in response to *S. typhimurium* infection from primary human monocytes depended on NLRP3. In response to *S. typhimurium* infection, IL-1β release occurred both with and without a LPS priming step (Fig. 3a,b). Priming did not affect the inhibition of IL-1β release by MCC950 (Fig. 3a,b). At both 1 h (Fig. 3a) and overnight (Fig. 3b) following *S. typhimurium* infection, there was a significant inhibition of IL-1β release when primary human monocytes were pre-treated with MCC950. *S. typhimurium* induced IL-1β release was also inhibited by high extracellular K^+^ at 1 h (Fig. 3c), but not after overnight incubation (Fig. 3d). MCC950 also caused a reduction in IL-1α release after overnight incubation (Fig. 3f), but not at the early time point (Fig. 3e). IL-6 release in response to *S. typhimurium* was independent of NLRP3 as MCC950 had no effect (Fig. 3g,h).

### LPS priming is not required for *S. typhimurium* induced IL-1β release

The majority of previous studies used LPS priming prior to *S. typhimurium* infection^22, 24^. However, as *S. typhimurium* is a Gram-negative bacterium its outer membrane contains LPS. LPS priming did not increase IL-1β levels at both early and late time points after *S. typhimurium* infection (Fig. 3a,b). Additionally, LPS priming did not affect the amount of IL-1α or IL-6 released (Fig. 3e–h). Overall, these data suggest that an additional LPS priming does not enhance NLRP3 activation in monocytes, in response to *S. typhimurium* after overnight incubation, with infection alone sufficient to induce priming and secretion of IL-1.

### Acute *S. typhimurium* infection does not cause pyroptosis in human primary monocytes

**Figure 4:**
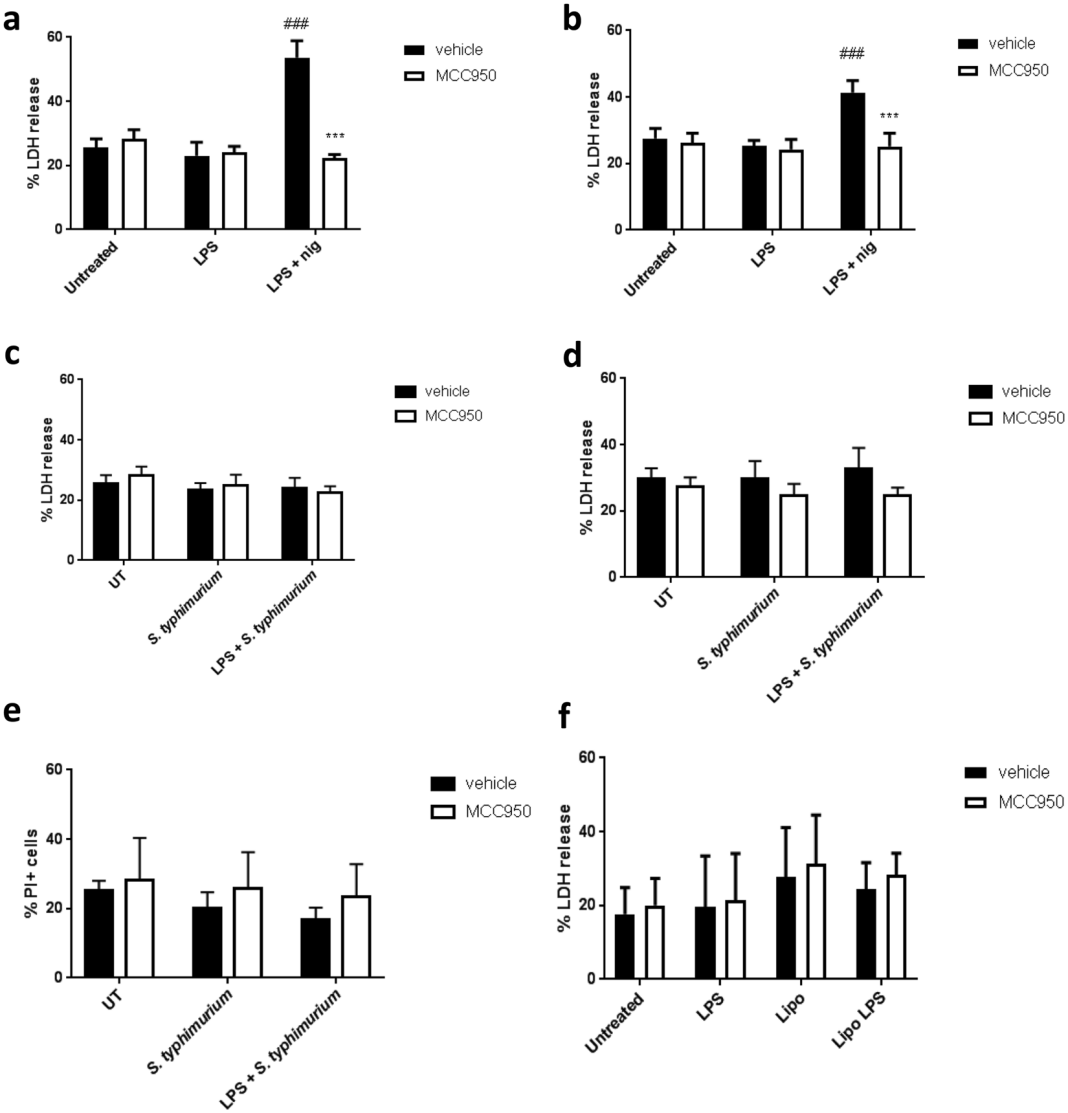
Acute *S. typhimurium* infection and intracellular LPS do not cause pyroptosis in human monocytes. (**a**,**b**) Primary human monocytes were either pre-treated with or without MCC950 (10 μM), and then stimulated with LPS (1 μg/ml) for 4 h. Nigericin (10 μM) or vehicle was added for 1 h after LPS incubation. Cell death was assessed using LDH assay on supernatants collected after nigericin treatment at 5 h (**a**) or overnight (**b**). (**c**,**d**) Primary human monocytes were either pre-treated with or without MCC950 (10 μM), and then incubated for 3 h with or without LPS (200 ng/ml). *S. typhimurium* (MOI 10) was added 1 h before addition of gentamycin and further incubation of either 1 h (**c**) or overnight (**d**). Cell death was assessed using LDH assays of the collected supernatant. *S. typhimurium* causes no increase in cell death either after 1 h or overnight incubation, confirmed by propidium iodide (PI) staining after overnight incubation (**e**). (**f**) Primary human monocytes were either pre-treated with or without MCC950 (10 μM), then LPS was transfected into the monocytes using Lipofectamine 2000 (Lipo LPS), or added extracellularly without Lipofectamine (LPS). Lipofectamine without LPS (Lipo) was used as a control. Data are presented as mean cytokine release ± s.e.m. Graphs (**a**–**c**) representative of four independent donors, graph (**d**) is representative of 5 independent donors, and graphs (**e**,**f**) representative of 3 independent donors, each performed in triplicate wells. ***P < 0.001 significant effect of MCC950 compared to vehicle group with matching stimuli. ^###^P < 0.001 significant induction of LDH release by stimuli compared to untreated control. Significance measured with a with-in subjects design two-way ANOVA followed by Holm-Šidák corrected post-hoc analyses.

MCC950 has been shown to inhibit cell death (as measured by the release of the intracellular enzyme lactate dehydrogenase (LDH) through a loss of membrane integrity) in BMDMs caused by LPS priming and nigericin treatment^22^. Therefore, we sought to determine if pyroptosis was induced following similar mechanisms in human monocytes. Thus, monocytes were pre-treated with MCC950 to assess the effect on cell death. Under these conditions, LDH release from the monocytes was increased when the cells had been treated with both LPS and nigericin, and this was inhibited by MCC950 at both the 5 h and overnight collection time-points (Fig. 4a,b). Treatment with LPS alone did not cause an increase in LDH release (Fig. 4a,b).

Recently, pyroptosis has been shown to be independent of the NLRP3 inflammasome when the non-canonical pathway is activated^29^. Therefore, monocytes were treated with or without MCC950 prior to infection with *S. typhimurium*. However, in response to *S. typhimurium* infection primary human monocytes showed no increase in LDH compared to untreated cells at both 1 h after infection (Fig. 4c) and after overnight incubation (Fig. 4d), regardless of LPS priming.

In addition to measuring LDH we also used propidium iodide (PI) staining to confirm that the monocytes remained viable after infection (Fig. 4e). Furthermore, there was no cell death in primary monocytes when the non-canonical pathway was activated by LPS transfected into the cytosol (Fig. 4f), consistent with the earlier observation that human primary monocytes show resistance to pyroptosis.

## Discussion

The association between pyroptosis and IL-1β and IL-1α release is a much discussed topic, yet it is still unclear how interrelated the two inflammasome-dependent processes are. Primary human monocytes are particularly intriguing as they have displayed unique pathways of inflammasome activation, differing from other immune cell types^14, 21^. This includes the ability to release IL-1β in response to LPS alone, instead of the two-step inflammasome activation pathway required in macrophages^14^. Here we sought to investigate the role of the NLRP3 inflammasome in both pyroptosis and IL-1 release in primary human monocytes during acute *S. typhimurium* infection.

NLRP3 and NLRC4 are suggested to play redundant roles in the clearance of *S. typhimurium* infection, so each can compensate for the other, resulting in reduced but not abolished inflammatory responses when either is knocked out^18^. It has also been shown that NLRC4 and NLRP3 can localise to the same inflammasome complex in response to *S. typhimurium* infection in non-LPS primed macrophages^16^. Recent studies have also reported that *S. typhimurium* does not depend on NLRP3 in BMDMs^30^ and *in vivo* ^27^, or that NLRP3 plays an accessory role during *S. typhimurium* infection in murine BMDMs, recruited by NLRC4 to amplify caspase-1 activation^28^. However, NLRP3 has no effect if the cells are not primed with LPS prior to infection. Here, although we have not ruled out an involvement of NLRC4, we have shown that there is an absolute requirement for NLRP3 for IL-1β secretion during acute *S. typhimurium* infection from primary monocytes. We have demonstrated the importance of NLRP3 for IL-1β secretion in response to *S. typhimurium* infection even when the monocytes were not primed with LPS. As monocytes are also able to release IL-1β in response to LPS alone, without a second stimulus, these data suggest that monocytes could already be inflammatory and ‘primed’, and caspase-1 has been reported to be constitutively active in human monocytes^21^ (although also see ref. 14).

Nigericin-induced IL-1α release is blocked by MCC950 in BMDMs, yet it has no effect on IL-1α release in mouse macrophages treated with monosodium urate crystals (activating the NLRP3 inflammasome)^22^, and it has previously been reported that IL-1α can be secreted through NLRP3 dependent and independent mechanisms depending on the stimulus^31^. In our study, secretion of IL-1α was dependent on NLRP3 during acute *S. typhimurium* infection, but did not require NLRP3 when the non-canonical pathway was activated by transfection of LPS. Our data therefore suggest that IL-1α is activated by multiple pathways. As IL-1α is one of the first cytokines to be released in response to infection, it could be that multiple pathways of activation ensure a maximal innate immune response in the early hours of infection, in case one route of IL-1α induction becomes blocked or is evaded by *S. typhimurium*.

The resistance to pyroptosis demonstrated by primary human monocytes in response to infection with *S. typhimurium* may offer added protection for the host, as by early-recruited monocytes maintaining their viability and not releasing intracellular contents; the pathogens are not released into the blood surrounding the tissue. This importantly limits sepsis and further endangering the host. Interestingly, it was recently reported that macrophages retain bacteria in a pore-induced intracellular trap so they are not released into the extracellular environment^32^. Our evidence suggests primary human monocytes do not employ this mechanism. Additionally, as a role of monocytes is to differentiate on localisation to tissue, undergoing pyroptosis before they reached this crucial stage could cause a reduction in the necessary macrophages and dendritic cells in peripheral tissues. As neutrophils have also been shown to be resistant to pyroptosis when challenged with *S. typhimurium* ^33^, it could be that the mechanism used for IL-1 release in circulating cells has evolved differently to the mechanisms in tissue resident cells such as macrophages. The lack of pyroptosis in monocytes may also be a result of the ability of *S. typhimurium* to evade being destroyed by monocytes, allowing them to survive and enable efficient dissemination of infection into multiple tissues.

These data shown here indicate that pyroptosis and IL-1β and IL-1α release in response to acute *S. typhimurium* infection are separable and can occur independently, despite both being regulated by caspase-1. These data also suggest that the activation of caspase-1 does not automatically lead to pyroptosis and suggests there is a further regulatory step for pyroptosis. As gasdermin D (GSDMD) has been identified as a substrate of caspase-1 and caspase-11, and causes the pores in the membrane leading to pyroptosis^25, 34^, it could be that in primary human monocytes there is an alternative mechanism of pyroptosis with different regulatory steps.

In summary, we have highlighted a difference between primary human monocytes and other immune cell types, by showing that efficient release of IL-1β and IL-1α during acute *S. typhimurium* infection is dependent on NLRP3 in primary human monocytes. Additionally, this study shows that the release of IL-1β in response to *S. typhimurium* infection occurs independently of pyroptosis. The ability of early recruited monocytes to release pro-inflammatory cytokines without undergoing pyroptosis may allow the cells to have a heightened and sustained immune response that is necessary to clear the host of infection.

## Methods

### Monocyte isolation

Human peripheral blood mononuclear cells (PBMCs) were isolated by Ficoll-Hypaque density gradient centrifugation (GE Healthcare) from buffy coats obtained from anonymous donors provided by the National University Hospital Transfusion Centre, Singapore. Ethical approvals for all blood sources and processes used in this study have been approved by National University of Singapore Institutional Review Board (license: NUS-IRB 12-044E). Subjects gave written informed consent in accordance to the Declaration of Helsinki. All experiments were carried out in accordance with the approved guidelines and regulations.

Isolation of monocytes was performed using negative selection (except Fig. 2c which used positive selection) with the Monocyte Isolation Kit II (Miltenyi Biotech), according to the manufacturer’s instructions. Monocytes were plated in tissue culture non-treated 48-well plates (2.5 × 10^5^/well/250 µl) in RPMI 1640-Glutamax media supplemented with 3% human serum (Gemini Bio product). Where the specific NLRP3 inhibitor MCC950 (10 µM; Sigma-Aldrich), or KCl (20 mM) were used, monocytes were either pre-treated for 30 min before the addition of *Escherichia coli* LPS (serotype O55:B5, Alexis Biochemicals, 1 µg/ml, 4 h). Nigericin sodium salt (10 µM; Sigma-Aldrich) was added 1 h before the collection of supernatants and lysates.

### Salmonella infection of monocytes

*Salmonella enterica* serovar Typhimurium (*S. typhimurium*)^35^ was cultured overnight on an LB agar plate LB broth. A single colony was picked and cultured overnight in LB broth with agitation at 37 °C. The liquid culture was then diluted 1:1000 in fresh LB broth and *S. typhimurium* was cultured until OD measured at 0.6–0.8 (mid log-phase). Aliquots of *S. typhimurium* were prepared by adding 1:10 80% glycerol to culture and stored at −80 °C. A sample of aliquots from the same batch were thawed and plated on LB agar to determine the concentration of viable bacteria.

For monocyte infection, aliquots of *S. typhiumurium* were thawed and washed twice in 500 µl PBS, then diluted to the appropriate multiplicity of infection (MOI) in antibiotic-free complete cell culture medium. Human primary monocytes were plated and treated or not with *Escherichia coli* LPS (serotype O55:B5, 200 ng/ml Alexis Biochemicals) for 3 h to prime the inflammasome. *S. typhimurium* was then added to the cells at MOI 10 and the plates were centrifuged for 5 min (400 × g). After 1 h, gentamycin (100 µg/ml) was added to kill extracellular *S. typhimurium* and ensure that only intracellular bacteria were present. Infected monocytes were either collected after 1 h or incubated overnight. Cell-free supernatants and lysates were collected.

### LPS transfection

Lipofectamine 2000 (Life Technologies) diluted in optiMEM (Gibco) was incubated for 5 min at RT and mixed with an equal concentration/volume of LPS (20 µg/ml) diluted in optiMEM. After 25 min incubation, lipofectamine/LPS mixture was added at 1:10 dilution to monocytes plated at 1 × 10^6^/ml in optiMEM. To block NLRP3 inflammasome activation, monocytes were also treated with MCC950 (10 µM) or KCl (20 mM) for 30 min prior to lipofectamine/LPS treatment. After 6 h antibiotic-free RPMI supplemented with 20% FBS was added to dilute the mixture by 50% (final FBS concentration 10% and volume 250 µl/well), before a further overnight incubation.

### Cytokine release

Cell-free supernatants were collected from monocyte cultures and IL-1α (R&D Duoset), IL-1β, and IL-6 (Biolegend) were determined by ELISA following the manufacturer’s instructions.

### Cell death assays

The presence of LDH in cell-free supernatants was measured using CytoTox 96® Non-Radioactive Cytotoxicity Assay (Promega), following the manufacturer’s instructions. Cell death was calculated as percentage of LDH released by total lysis. Cell death was also determined by Propidium Iodide (PI) staining using flow cytometry. Treated monocytes were stained with PI (2 µg/ml; Sigma-Aldrich) in FACS buffer (1% BSA, 0.01% Sodium Azide) immediately before being analysed on a LSRII flow cytometer (BD Biosciences). Unstained monocytes and cells containing 0.1% triton were used as controls. Data were analysed using FlowJo (Treestar).

### Statistical analysis

All statistical analysis was performed using GraphPad Prism 7 and R v3.3.0 for Windows^36, 37^. Data were analysed with a two-way ANOVA using a within subject design followed by Holm-Šidák corrected post-hoc analyses. Homoscedasticity and normality of the residuals were assessed graphically using residuals vs fitted and Q-Q plots, respectively. Appropriate transformations were performed when necessary.
